# Supplementary material for: Suitability of just-in-time adaptive intervention in post-COVID-19-related symptoms: A systematic scoping review
Source: PLOS Digit Health. 2025 May 29;4(5):e0000832. doi: 10.1371/journal.pdig.0000832 (PMC12121805; doi:10.1371/journal.pdig.0000832)
Supplement: S1 Table — Data of included studies. Extracted JITAI key components, evaluation, and barriers of included articles. (DOCX) [file pdig.0000832.s002.docx]

**S1 Table**. *JITAI key components, evaluation, and barriers of included articles*

| **Author (year)** | **Intervention option** | **Tailoring option** | **Decision rules** | **Effectiveness/ feasibility** | **Barriers** |
| --- | --- | --- | --- | --- | --- |
| ***Fatigue and pain symptom cluster*** | | | | | |
| Azizoddin et al., 2024 | Supportive messages with pain-CBT education on pain, mood, pain catastrophising, and sleep disturbance; recommendations to contact care team or emergency services | Once daily EMA on pain severity and interference, mood, pain catastrophising, and sleep quality;  Once weekly EMA on symptom severity (e.g. fatigue), opioid analgesic use and relief, pain acceptability, and progress towards self-identified goals | Static: messages were always sent, but tailored to level of variable: e.g. high pain scores prompted a related message and content, such as advice on relaxation;  Following the weekly EMA, goal attainment and opioid use were checked | Acceptability:  87% of acceptability items (understandability, ease of use, satisfaction, enjoyability, and time to complete) were rated ≥ 4/5 (high acceptability)  Feasibility:  73% completed ≥ 50% and 27% completed ≥ 75% EMA, indicating high feasibility. However, education links in JITAI were only used by few participants | Some participants found the EMA repetitive. Many were frustrated that the EMA expired an hour after they were pushed. Although the tailored messages were considered appropriate and helpful, participants did not want to review the educational content included, as they were perceived as burdensome |
| Hiremath et al., 2019 | PA monitoring and feedback on goal attainment; recommendation push messages to start PA; reinforcement messages to continue PA | Continuous measures of PA (sensed by accelerometer and wheelchair rotation monitor) to classify moderate-to-vigorous PA via decision tree ML;  Personalised goals based on patterns of PA of previous day | Static: at least 3 minutes of estimated moderate-to-vigorous PA for push reinforcement notifications.  Check whether personalised daily goal was attained. | Acceptability: high  Compliance: high Effectiveness: significant increases in low and moderate-to-vigorous PA in 11 out of 16 of participants. | Minor technological issues, primarily with the app or connectivity.  Participants disliked a lack of control over prompting, tracking of activities, and notifications.  JITAI did not account for all contexts, including chronic pain or hospitalisation. |
| ***Psychological problems symptom cluster*** | | | | | |
| Bell et al., 2023 | Delivery of CBT micro-interventions with 12 variations | EMA on repetitive negative thinking, mood, activities and location,  randomly 3 times a day between 9 AM and 9 PM | Static: tailored recommendations for CBT micro-interventions were based on a preprogrammed algorithm that matches strategies to the level of repetitive negative thinking, (high or low), mood (positive or negative), location (at home or away from home), and activity (active or passive). | Acceptability: generally positive, with all scores (engagement, functionality, aesthetics, information, app quality, and perceived impact) >3.5/5 and 96% of participants would recommend the app to others  Feasibility: target sample size easily reached within 6 months; 59% retention for 6 weeks (high)  Preliminary effectiveness:  Compared to the control group, JITAI group reported significant decreases in anxiety (Cohen *d* = 0.61) and repetitive negative thinking (Cohen *d* = 0.87) over time. No significant differences over time for depression, but group differences in favour for the JITAI group (Cohen *d* = 0.50) | No dynamic tailoring integrated yet; ML and passive sensing might improve the effectiveness of the intervention. |
| Beltzer et al., 2022 | N/A | Time of day was continuously included.  EMA measured semi-randomly 6 times per day: context, motivation to change ER strategies. ER strategy use, perceived ER effectiveness, and post-ER affect observed. | Static: Contextual bandit: “we observed how participants reported regulating their emotions and their subsequent affect” | N/A | N/A |
| Carlozzi et al., 2022 | Push notification to promote health behaviours (PA and sleep hygiene) and improve mood (anxiety, depression, strain); data feedback (monitoring); education (facts and tips); support messages.  Notification content was randomly drawn from a pool of over 400 messages. | Continuous accelerometer-based estimates of physical activity and total sleep time (FitBit).  Message content was personalised based on data.  EMA on health-related quality of life (caregiver strain, depression, anxiety) once daily | Static: 50/50 chance of receiving a personalized push notification each day | Effectiveness (exploratory; not enough statistical power): JITAI significantly better than control at 3-months for strain, anxiety, depression, and sleep-related impairment.  Compliance: high  Acceptability: high | Worse symptom severity (i.e. greater levels of depression and fatigue and lower positive affect) was associated with worse compliance for EMA.  Not all subgroups were enthusiastic about the FitBit and study for unknown reasons. Not all participants felt comfortable with or confident in using FitBit.  Sample not big enough for effectiveness and generalisation of findings. |
| Jacobson & Chung, 2020 | N/A | Direct location (GPS or Wi-Fi), type of location, local weather, heart rate (averages and variability) and number of outgoing phone calls, passively measured once per hour.  EMA on depressed mood (sadness and loneliness) once per hour) | Static: ML algorithms used to predict chance of depression based on past 24 hours of sensor data (as part of a retrospective observational study).  Modelling was based on both between-person and within-person variability; the latter weighting stronger. | High correlation between predicted depressed and observed mood scores. | N/A |
| Pulantara et al., 2018b | Delivery of sleep education and personalised sleep tips; monitoring | EMA on self-reported sleep time and quality; lifestyle behaviours (e.g. caffeine and alcohol consumption and number and duration of daytime naps) once daily; EMA on anxiety and depression once weekly. | Static: manually triggered by clinician based on self-reported input | Rated as highly usable | Technical issues with server outage. FitBit not sensitive enough to measure sleep. |
| Pulantara et al., 2018a | Delivery of personalised CBT for insomnia: bedtime reminders, wake-up alarms, bibliotherapy | EMA on self-reported sleep time and quality; lifestyle behaviours (e.g. caffeine and alcohol consumption and number and duration of daytime naps) once daily; EMA on anxiety and depression once weekly.  Sleep data via accelerometer (FitBit) | Static: manually triggered by clinician based on self-reported and passively sensed sleep data | Significant improvement in sleep, depression, anxiety and PTSD symptoms with moderate to large effect sizes (Cohen *d* = 0.57 – 1.93); 74 – 82% of participants clinically improved | Not clear whether this JITAI is suitable for persons with severe psychiatric disorders or sleep apnoea |
| Ren et al., 2023 | N/A | Smartphone sensing data, namely PA (accelerometer), phone usage, sleep, location, time of day. | N/A | Personalised predictions using ML are best to predict heightened states of anger, sadness, and nervousness using smartphone sensing data | Type of smartphone-derived variables can lead to alternative (possible improved) predictors; big sample sizes are necessary for improved within-subject ML |
| Wahle et al., 2016 | Delivery of CBT micro-interventions with 80 variations | Smartphone sensing data, including time of day, location (Wi-Fi and GPS), PA (accelerometer), and social activity including smartphone usage | Context information, user preference and decision logics were used to recommend micro-interventions; at first static (predefined), later dynamic (based on user behaviour). For example, lack of general activity would result in an increased probability for a recommendation to take a walk. | For participants with a clinically relevant depression score (PHQ-9) and an extended MOSS app adherence, a significant drop in depressive symptoms (measured by PHQ-9) was observed.  Supervised ML models can be used to predict depressive state | Low motivation for adherence can be an issue |
| Wang & Miller, 2023 | Delivery of rumination-focused CBT, specifically training in problem-solving, conflict attribution, and emotion regulation; supportive or motivating messages | EMA every 3 hours on stressful events (presence and nature) in the past 3 hours that can trigger rumination episodes; type of activities (i.e. whether they could engage in an intervention) | Static: if a trigger for a ruminative episode was encountered, training materials that best targeted the trigger of their ruminative episode were provided | Compared to the control group, the intervention group reported a significantly greater reduction in number of rumination episodes (Cohen *d* = 2.5) and significantly reduced time spent in rumination (Cohen *d* = 1.84)  No time-lagged effects by (e.g.) negative emotions or previous rumination experience on next rumination episodes for intervention group (unlike control group), supporting the evidence of the JITAI aspect of the CBT intervention | None mentioned for the JITAI directly, but substantial delays in gaining ethical approval for the study were described |

*Notes*. CBT = cognitive behavioural therapy; EMA = ecological momentary assessment; ER = emotion regulation; JITAI = just-in-time adaptive intervention ML = machine learning; N/A = not applicable; PA = physical activity
