# Supplementary material for: Suitability of just-in-time adaptive intervention in post-COVID-19-related symptoms: A systematic scoping review
Source: PLOS Digit Health. 2025 May 29;4(5):e0000832. doi: 10.1371/journal.pdig.0000832 (PMC12121805; doi:10.1371/journal.pdig.0000832)
Supplement: Attachment — Submitted filename: Response to reviewers.docx [file pdig.0000832.s004.docx]

Dear editor, dear reviewers,

Thank you for your time and for inviting us to revise our manuscript, *Suitability of just-in-time adaptive intervention in post-COVID-19-related symptoms: A systematic scoping review*. We have outlined below our responses (in blue) to the comments by the reviewers and the editor.

**Responses to the reviewers**

***Reviewer #1***

The statistical analysis has been provided appropriately. Author has provided all the data underlying the findings and is written in standard english. The manuscript can be accepted for publication with minor revisions.

We thank the reviewer for these comments. As it was not specified what should be revised, we have gone over the manuscript again to check for any possible spelling and reference mistakes and unclarities.

***Reviewer #2***

COVID-19 was and has been a worldwide systemic shock to the healthcare system. Curiously what has emerged has been a post COVID-19 syndrome which has a myriad of symptoms and signs, is difficult to diagnose and requires adaptive strategies to enhance diagnosis, treatment and generally manage these often-difficult patients. Just in time adaptive interventions is a strategy allowing rapid course correction in the approach to patients. In this scoping review authors screened 341 papers and identified 11 which were suitable for analysis through a “just in time lens.” The search terms utilized included symptom clusters of fatigue and pain, respiratory problems, cognitive dysfunction and psychological problems. The end points examined were those related to just in time interventions including intervention options, tailoring variables and decision rules. All in all, the authors found that in the limited number of papers identified - 11 - two articles addressed fatigue or pain and nine address psychological problems. Curiously no papers were found which addressed more primary medical issues such as respiratory complications or cognitive dysfunction. The authors conclude that just in time strategies are acceptable and feasible though with limited evidence supporting this plausible conclusion, and further go on to endorse it just in time approach when needed for rapid decision making. While this review covers much ground there are several issues which could be addressed to strengthen the paper and are discussed below.

We thank the reviewer for this extensive summary of the study and all comments. After reflection, we understand that the scope of the study was not made sufficiently clear, namely that just-in-time adaptive interventions (JITAIs) generally aim to support patient self-management and self-regulation. This was also our scope in the context of real-time support in post-COVID-19 symptom management. In line with this, below we discuss below per remark how we aimed to clarify this in the manuscript to aid readers in the interpretation of this study.

While the review has covered and states up front “200 symptoms have been described in post COVID syndrome,” the authors then go on to examine a limited number of these. In light of the fact that out of 340 papers only 11 are presented and overall we are dealing in this paper with limited number of data points It would be useful to include a table, beyond the reference of the symptoms. While authors or others may say this is beyond the scope of the review, in light of the limited data in the review this would be a useful addition for the reader to get a better feel for the symptoms involved. If this is viewed as being too extensive perhaps the top 100 or 50 symptoms could be provided this would be a useful addition to the reader and would increase the value of the paper for a broader readership.

Thank you for this remark. We appreciate that an overview of all (or many) symptoms can be insightful in that it brings attention to post-COVID-19 syndrome to readers unfamiliar with this disease. However, as it is a very complex syndrome potentially affecting multiple organ systems, we think that providing just a list of symptoms would do a disservice to readers, as systematic reviews would do and have done a much better job of reporting what is actually going on with this syndrome. We would therefore advise interested readers to look up the cited reviews, for example those by Davis et al. (2023) and Greenhalgh et al. (2024). There are also great meta-analyses and other systematic reviews that focus on the prevalence of post-COVID-19 symptoms. See for example the below figure by Lopez-Leon et al. (2021).

Furthermore, we argue that providing such an overview of symptoms that do – or most likely may – not benefit from what JITAI aim to do, might confuse readers. As we will now explain more explicitly in our manuscript, while some symptoms are best served by biomedical treatment and may not require a lot of self-management by the patient, the most commonly reported long-term persistent symptoms (fatigue, pain, breathlessness, cognitive dysfunction/brain fog and psychological problems) require much more self-regulation, such as energy management through pacing. Consequently, they may possibly be supported through JITAI.

In line with this discussion, we have added a paragraph on symptoms and post-COVID-19 treatment on pp. 4 and 5 of the revised manuscript and made some other minor adjustments to draw attention to the self-management aim.

Source: Lopez-Leon S, Wegman-Ostrosky T, Perelman C, Sepulveda R, Rebolledo PA, Cuapio A, et al. More than 50 long-term effects of COVID-19: a systematic review and meta-analysis. Sci Rep. 2021;11(1):16144. <https://doi.org/10.1038/s41598-021-95565-8>.

Relatedly while the paper focuses on symptom complexes it would be useful to address and provide a table of other signs and lab or imaging abnormalities in post COVID-19 syndrome. While one may say this is beyond the scope of the paper and relates to other reviews, having things immediately present while one reads a review like this enhances the insight and broad perspective and value for a wide readership. This should be strongly considered.

Regarding this comment too, we appreciate the reviewer’s advice to provide an overview of post-COVID-19 syndrome. In line with this request, we have added a paragraph of possible signs and laboratory and imaging abnormalities that can be used for diagnosis and monitoring. As the reviewer has pointed out, diagnosing post-COVID-19 is difficult. Because of the huge range of possible assessment options, which depend on the patient’s symptoms and functional status (and medical history), we must refer to a review by biomedical experts for a more nuanced and thorough discussion (e.g. Yan et al., 2023). We also like to point out that these signs and abnormalities are unlikely to be suitable for JITAI, because they are impractical or impossible to include into the tailoring variable system and may not be relevant for real-time self-management support.

Similar suggestion applies to just in time interventions. It would be useful to add a figure diagramming how a just in time intervention works. While the authors have nicely provided a sentence or two related to smoking cessation and more general figure would enhance the value for a broad readership. Relatedly, they could bring in in that figure the key components related to intervention options tailoring variables decision rules and decision points this would make it more interpretable to others less familiar with this technology.

Definition and working understanding of just in time interventions. It is only at the end of the paper that in the discussion it is called out that there's no consensus on definitions of just in time interventions. While the reviewer understands that this is a review on just in time interventions for specific COVID related situation a bit of a deeper discussion about what just in time interventions are, what is their scope? how do they work? what are the pros and cons? would be useful to enhance the value for the broader readership. This should be placed both up front as well as in the discussion.

Similarly adding to the figure images related to wearables and how information enters and exits a JITAI strategy would be useful

Thank you for these excellent suggestions, reviewer. We have added a new Figure 1 that provides an overview of the key components and potential input methods of JITAIs. This should help readers understand better how these systems generally work. Additionally, we have amended the first paragraph on JITAIs (pp 6-7) to extend on how JITAIs generally aim to support self-management, and have clarified their pros and cons.

It would be very useful to include a table of abbreviations. While there are many complex terms introduced and abbreviations are then cited, as this article is long one often needs to go back to identify what these abbreviations are. For those readers unfamiliar with this area of work finding things such as PEM and others in one location in a table would be useful.

Thank your for pointing this out. We have now included a list of abbreviations at the end of our manuscript.

Note to the editor: we were unsure where such as list should be placed and defer to your judgement.

It would be very useful to address the utility of just in time interventions for more substantive signs and laboratory abnormalities associated with post COVID syndrome. While it is understood that this is a vague syndrome and the paper addresses symptom complexes the utility of this with an eye to the future, i.e. aimed at data provided by periodic lab tests or wearables even more dynamically, could have impact on more organic involvements in post COVID syndrome. The authors could at least provide their vision as to how this might be useful - to again add more data or perspective to this review.

We thank the reviewer for asking us to clarify this. Unfortunately, we do not think that including these types of test are beneficial or sensible for JITAI systems. As discussed previously, and what should be clearer now in the text, JITAIs aim to support self-management through health behaviours. As a result, JITAIs aim to assess either (parts 0f) these behaviours, user experiences (e.g. fatigue severity; current mood) or proxies of either of these (biomarkers such as heart rate (variability); step counts; GPS location; et cetera). We are not aware of any laboratory or imaging tests that would result in a *just-in-time* intervention. Although we would very much like to see a (bigger) role of digital health technologies for *clinical* management, such as monitoring post-COVID-19 signs and symptoms (i.e. telemonitoring; see for some examples and ideas Romaszko-Wojtowicz et al. 2022 (<https://doi.org/10.3390/ijerph19095268>) and 2023 (<https://doi.org/10.3390/medicina59091541)>), we think this is too far beyond the scope of our review on JITAIs and the focus on self-management.

**Responses to the editor**

1. Please amend your detailed Financial Disclosure statement. This is published with the article. It must therefore be completed in full sentences and contain the exact wording you wish to be published.

i. Please clarify all sources of funding (financial or material support) for your study. List the grants (with grant number) or organizations (with url) that supported your study, including funding received from your institution.

ii. State the initials, alongside each funding source, of each author to receive each grant.

iii. State what role the funders took in the study. If the funders had no role in your study, please state: “The funders had no role in study design, data collection and analysis, decision to publish, or preparation of the manuscript.”

iv. If any authors received a salary from any of your funders, please state which authors and which funders.

We have amended the Financial Disclosure statement as follows:

‘GS was financially supported by the foundation ‘Friends of MST’ (Dutch: stichting Vrienden van MST; <https://www.samenvoormst.nl/?locale=en>), which through donations supports projects and initiatives to improve healthcare and health practices within and beyond Twente, the Netherlands. BB and CB have no funding to declare. The funders had no role in study design, data collection and analysis, decision to publish, or preparation of the manuscript.’

2. Please make sure the funding information on the submission form matches your financial disclosure statement. Please indicate by return the full and correct funding information for your study and confirm the order in which funding contributions should appear. Please be sure to indicate whether the funders played any role in the study design, data collection and analysis, decision to publish, or preparation of the manuscript.

The funding information on the submission form should now match the financial disclosure statement. All information is correct and in the right order:

Only GS was financially supported by the foundation ‘Friends of MST’ (Dutch: stichting Vrienden van MST). The funders had no role in study design, data collection and analysis, decision to publish, or preparation of the manuscript.

3. Please send separate figure files in .tif or .eps format. Also, remove the figures from your manuscript file but keep the legends.

For more information about figure files please see our guidelines:

https://journals.plos.org/digitalhealth/s/figures

https://journals.plos.org/digitalhealth/s/figures#loc-file-requirements

We have removed the figure from our manuscript, and after checking the .tif files with the PACE tool, have uploaded it and the newly added figure separately from the manuscript.

4. We have noticed that you have uploaded Supporting Information files, but you have not included a list of legends. Please add a full list of legends for your Supporting Information files after the references list.

We have now included a legend of the Supporting Information files and placed it after the references list.

5. We notice that your supplementary Table A1 are included in the manuscript file. Please remove them and upload them with the file type 'Supporting Information'. Please ensure that each Supporting Information file has a legend listed in the manuscript after the references list.

We have removed this table and uploaded it as Supplementary File 2. A legend has been added after the references list.

6. We note that your Data Availability Statement is currently as follows: “N/A (however, data from studies included in this systematic scoping review is included as an appendix)”

Please confirm at this time whether or not your submission contains all raw data required to replicate the results of your study. Authors must share the “minimal data set” for their submission. PLOS defines the minimal data set to consist of the data required to replicate all study findings reported in the article, as well as related metadata and methods (https://journals.plos.org/plosone/s/data-availability#loc-minimal-data-set-definition).

All data used for this study were indeed in the appendix. To clarify, we have amended the Data Availability statement as follows:

‘All data required to replicate the results of this study are included in SI File S2’
